# In healthy volunteers, taking flucloxacillin with food does not compromise effective plasma concentrations in most circumstances

**DOI:** 10.1371/journal.pone.0199370

**Published:** 2018-07-12

**Authors:** Sharon J. Gardiner, Philip G. Drennan, Ronald Begg, Mei Zhang, Jared K. Green, Heather L. Isenman, Richard J. Everts, Stephen T. Chambers, Evan J. Begg

**Affiliations:** 1 Department of Infectious Diseases, Christchurch Hospital, Christchurch, New Zealand; 2 Department of Clinical Pharmacology, Christchurch Hospital, Christchurch, New Zealand; 3 Pharmacy Services, Christchurch Hospital, Christchurch, New Zealand; 4 Department of Medicine, University of Otago-Christchurch, Christchurch, New Zealand; 5 Toxicology, Canterbury Health Laboratories, Christchurch, New Zealand; 6 Department of Medicine, Nelson Hospital, Nelson, New Zealand; 7 Department of Pathology, University of Otago-Christchurch, Christchurch, New Zealand; TNO, NETHERLANDS

## Abstract

It is usually recommended that flucloxacillin is given on an empty stomach. The aim of this study was to compare total and free flucloxacillin concentrations after oral flucloxacillin, given with and without food, based on contemporary pharmacokinetic and pharmacodynamic targets. Flucloxacillin 1000 mg orally was given to 12 volunteers, after a standardised breakfast and while fasting, on two separate occasions. Flucloxacillin concentrations over 12 hours were measured by liquid chromatography-tandem mass spectrometry. Pharmacokinetic parameters, and pharmacodynamic endpoints related to target concentration achievement, were compared in the fed and fasting states. For free flucloxacillin, the fed/fasting area under the concentration-time curve from zero to infinity (AUC_0-∞_) ratio was 0.80 (p<0.01, 90% CI 0.70–0.92), the peak concentraton (*C*_max_) ratio 0.51 (p<0.001, 0.42–0.62) and the time to peak concentration (*T*_max_) ratio 2.2 (p<0.001, 1.87–2.55). The ratios for total flucloxacillin concentrations were similar. The mean (90% CI) fed/fasting ratios of free concentrations exceeded for 30%, 50% and 70% of the first 6 hours post-dose were 0.74 (0.63–0.87, fed inferior p<0.01), 0.95 (0.81–1.11, bioequivalent) and 1.15 (0.97–1.36, fed non-inferior), respectively. Results for 8 hours post-dose and those predicted for steady state were similar. Comparison of probability of target attainments for fed versus fasting across a range of minimum inhibitory concentrations (MICs) were in line with these results. Overall, this study shows that food reduced the AUC_0-∞_ and *C*_max_, and prolonged the *T*_max_ of both free and total flucloxacillin concentrations compared with the fasting state, but achievement of free concentration targets associated with efficacy was in most circumstances equivalent. These results suggest that taking flucloxacillin with food is unlikely to compromise efficacy in most circumstances.

## Introduction

Flucloxacillin is used in many parts of the world for treating infections due to methicillin- susceptible *Staphylococcus aureus* and *Streptococcus pyogenes*. It has a narrow spectrum of antimicrobial activity, is inexpensive, and is available in oral and intravenous formulations. Administration is complicated by the requirement for frequent dosing, usually four times daily. Compliance with oral administration is further compromised by the widespread recommendation that flucloxacillin should be taken on an empty stomach [1–4]. This may be associated with more upper gastrointestinal side effects and lead to some patients omitting doses around meal times and may be virtually unachievable in small children who often have variable eating patterns.

The advice to take flucloxacillin in a fasting state is based on studies conducted in the 1970s, and warrants reconsideration in light of the demonstration that the primary determinant of bacterial killing for β-lactam antimicrobials is the time above the minimum inhibitory concentration (MIC). The first published pharmacokinetic study of flucloxacillin showed that administration of flucloxacillin after a ‘standard breakfast‘ (undefined) to healthy volunteers delayed flucloxacillin absorption (peak at 2 hours for fed versus 1 hour for fasting) and reduced peak serum concentrations (of total drug) by ~40% compared with the fasting state (7.9 versus 12.6 mg/L, respectively, after a 500 mg oral dose) [5]. A subsequent study using the same dose had similar findings, with mean peak total serum concentrations of 6.2 mg/L at 3 hours after administration with food and 11.4 mg/L at 1 hour after administration on an empty stomach [6]. In both studies, blood was sampled for 6 hours after a single dose [5,6]. In a third study, patients (rather than healthy volunteers) receiving oral flucloxacillin every 8 hours therapeutically, administered after food, had mean peak serum concentrations 25–30% lower than when taken fasting [7]. Collectively, these studies indicated that food decreased the area under the concentration-time curve (AUC) and the maximum concentration (*C*_max_)_,_ and prolonged the time to maximum concentration (*T*_max_) of the concentration-time curve of total flucloxacillin. These findings have been interpreted to mean that efficacy would be greater in the fasted state. The data in the original publications cannot be accurately re-evaluated according to current pharmacodynamic end points as free flucloxacillin concentrations (i.e. appropriate for comparison against MICs) were not measured directly, and sampling times were short.

While it is clear that for β-lactam antibiotics the free time that concentrations exceed the MIC of the infecting organism over the dose interval (i.e. *fT*_>MIC_) is more predictive of success than peak concentrations [8], the precise pharmacokinetic/pharmacodynamic targets for flucloxacillin against *S*. *aureus* and *S*. *pyogenes* have not been fully determined. Evidence from both *in vitro* and animal studies indicate that for bacteriostasis, an *fT*_*>*MIC_ of 25–30% of the dosing interval is required for *S*. *aureus* and approximately 40% for streptococci [8,9]. An *fT*_*>*MIC_ of 50–60% of the dosing interval is required for maximum bacterial killing of staphylococci and 90–100% for streptococci [8,10]. An *f*T_>MIC_ target of approximately 50% has been used in previous studies investigating pharmacokinetic-pharmacodynamic endpoints of flucloxacillin dosing for near-maximum bactericidal effect against *S*. *aureus* [11]. The clinical relevance of targeting maximum kill versus bacteriostasis has not been rigorously tested across a variety of clinical settings, but the available data suggest that maximum killing is preferred in more severe settings and stasis may be all that is required for less severe infections in non-neutropenic hosts [9]. A flatter concentration-time curve where the time to maximum concentration is delayed, as occurs when an oral drug is taken with food, may have advantages for achieving *fT*_>MIC_ targets (e.g. 50% of the dose interval).

The aims of this study were to examine the effect of food on flucloxacillin concentrations utilising a sensitive assay to measure both total and free flucloxacillin over a full concentration-time curve, and to compare the results in terms of contemporary pharmacokinetic and pharmacodynamic parameters.

## Methods

### Study design

This was a prospective, open-label, two-way, cross-over study in 12 healthy non-smoking adult volunteers given flucloxacillin 1000 mg orally on two study days separated by a 7-day ‘washout’ period. The study took place at a dedicated research facility in Christchurch Hospital (Christchurch, New Zealand). Ethical approval was provided by the Northern B Health and Disability Ethics Committee. Written, informed consent was obtained from all volunteers. The research was registered as a clinical trial with the Australian New Zealand Clinical Trials Registry (ACTRN12617001046392).

Prior to the study each volunteer was screened by questionnaire, medical examination and blood testing to ensure there was no food intolerance, pregnancy, chronic illness, renal impairment (estimated GFR < 80 mL/min/1.73 m^2^) [12], liver dysfunction, malabsorption, or known intolerance of flucloxacillin. Volunteers were excluded if they took any regular medication (other than a hormonal oral contraceptive pill) or were intolerant of fasting for up to 10 hours.

On the first study day, volunteers were randomized so that half (n = 6) received the flucloxacillin after a high-fat high-calorie breakfast (fed state) and half received flucloxacillin on an empty stomach (fasting state). This order was reversed on the second study day. Prior to administration of flucloxacillin on the study days, volunteers were not allowed food for 8 hours but had free access to water. On the fasting study day water was all that could be consumed until two hours after receiving flucloxacillin, after which food was permitted. On the fed study day, each volunteer ingested a standardized high-fat high-calorie meal comprised of croissants 75 g, streaky bacon 50 g and cheddar cheese 50 g, equating to ~968 kcal of energy, 38 g of protein, 65 g of fat and 58 g of carbohydrate, meeting FDA recommendations for fed bioequivalence studies [13]. Caffeine was permitted only after the 4-hour blood sample and alcohol was forbidden until completion of the study day.

At the start of each study day, an intravenous catheter was inserted, baseline blood samples were taken and the catheter was flushed with 5 mL of sodium chloride 0.9%. Flucloxacillin was then given as 2 x 500 mg capsules (Staphlex®, Mylan, Auckland, New Zealand) with 240 mL of tap water. Timed blood samples were drawn into 2 x 4.5 mL EDTA tubes at 0.5, 1, 1.5, 2, 3, 4, 6, 8 and 12 hours after taking flucloxacillin, after the initial 5 mL of blood withdrawn from the catheter was discarded. Blood samples were centrifuged promptly and the resultant plasma was stored at -80°C until analysis for total and free flucloxacillin concentrations. All urine was collected from 0 to 12 hours after taking flucloxacillin, the total urine volume recorded and an aliquot retained for determination of total flucloxacillin concentrations.

### Assay and analysis

Total and free concentrations of flucloxacillin were analysed using a simple and validated LC-MS/MS method [14]. Standard curves were linear over the concentration range 0.2 to 100 mg/L (r > 0.99) in plasma and 0.005 to 10 mg/L (r > 0.99) in plasma ultrafiltrate. Intra- and inter-day coefficients of variation were < 10% over these concentration ranges. The lower limit of quantification was 0.2 mg/L for total flucloxacillin and 0.005 mg/L for free flucloxacillin.

Non-compartmental pharmacokinetics of total and free flucloxacillin concentrations were calculated for each volunteer in each treatment phase using PKSolver on the Microsoft Office 2010 Excel platform [15]. AUC from 0 h until infinity (AUC_0–∞_) was assessed using the linear up/log down trapezoidal rule, with extrapolation to infinity using C_last_/k, where C_last_ is the last measured concentration and k is the elimination rate constant. *C*_max_ and *T*_max_ were read directly from the raw data. The apparent oral CL (CL/F, where F = oral availability) was calculated from Dose/AUC_0–∞_. The half-life (*t*_1/2_) was calculated from 0.693/k. The protein binding was calculated using 1 –AUC_free_/AUC_total_. Statistical analyses were performed using Graphpad Prism for Windows (Version 6.05, July 7, 2014). The change in value of the pharmacokinetic parameters between the two regimens was compared using the paired Student’s t-test, with p<0.05 considered significant, and bioequivalence criteria (i.e. bioequivalence = mean ± 90% CI of the ratio fed/fasting within 0.8 to 1.25, and non-inferiority = mean ± 90% CI of the ratio fed/fasting > 0.8) applied if no difference was demonstrated. Analysis of AUC_0–∞,_ *C*_max_ and CL/F was based on geometric means, as is standard for bioequivalence studies.

The major pharmacodynamic end-point for comparison between fed and fasting was based on the *f*T_>MIC_. Specifically this was the free plasma flucloxacillin concentrations that exceeded each of 30%, 50% and 70% of 6 and 8 hours (representing likely dose intervals). These concentrations were read directly from the raw concentration-time curves constructed for each individual in the fed and fasting states using linear interpolation between data points in the ascending phase and log-linear interpolation in the descending phase.

### Modelling

Prediction of steady-state free concentrations was carried out as follows. Various models were considered based on visual examination of the raw concentration-time data for individuals, and for the amalgamated data using Monolix (lixoft.com/products/monolix). Monolix uses nonlinear mixed effects modelling to estimate mean values of the parameters for the population as well as inter-individual and inter-occasion (fed vs fasted) variability.

*A priori* the expected model for oral flucloxacillin was a one-compartment model with first-order absorption [16]. The delaying effects of food on absorption, noted above, raised the possibility of a *T*_lag,_ and either an altered absorption rate constant (*k*_a_) with first-order absorption or a different mechanism of absorption such as zero-order. The different models assessed therefore included zero-and first-order absorption with or without *T*_lag_, and one- and two-compartments. Proportional error was assumed.

Starting values for the iterative estimation of the pharmacokinetics of free flucloxacillin were approximated from mean literature values based on total concentrations in patients with normal renal function [11], corrected for assumed protein binding of 0.95 [17], and an oral availability (F) of 0.5 [16]. The starting values chosen for the one-compartment model with *T*_lag_ and first-order absorption were *T*_lag_ of 0.5 h, *k*_a_ of 2.5 h^-1^, apparent oral volume of distribution where F is oral availability (V/F) of 900 L, and *k*_e_ of 0.5 h^-1^. In the absence of previous data an estimated value of 2 h was used for the estimate of zero order absorption. The parameter estimates and their distributions for the chosen model were used to generate sample steady-state concentration-time curves by simulating 15,000 individuals in the fed and fasting states. From these curves estimates were made of free concentrations exceeding 30%, 50% and 70% of 6- and 8-hour dose intervals. The ratiosfor the steady-state concentrations in the fed and fasting states were then compared as stated above for raw concentrations.

The chosen model was then used to model the probability of target attainment (PTA) of free flucloxacillin concentration above MIC at steady-state over a range of different MIC values for 30%, 50% and 70% of 6- and 8-hour dose intervals. To aid interpretation of these PTA results, internationally published MIC_90_ values for the bacteria commonly treated with flucloxacillin i.e. *S*. *pyogenes* (0.1 mg/L) and *S*. *aureus* (0.5 mg/L) [5,18,19] are provided. EUCAST tables contain no MICs for flucloxacillin (www.eucast.org). The MIC_50_ is also provided for *S*. *aureus* (0.25 mg/L) [5].

## Results

**Fig 1:**
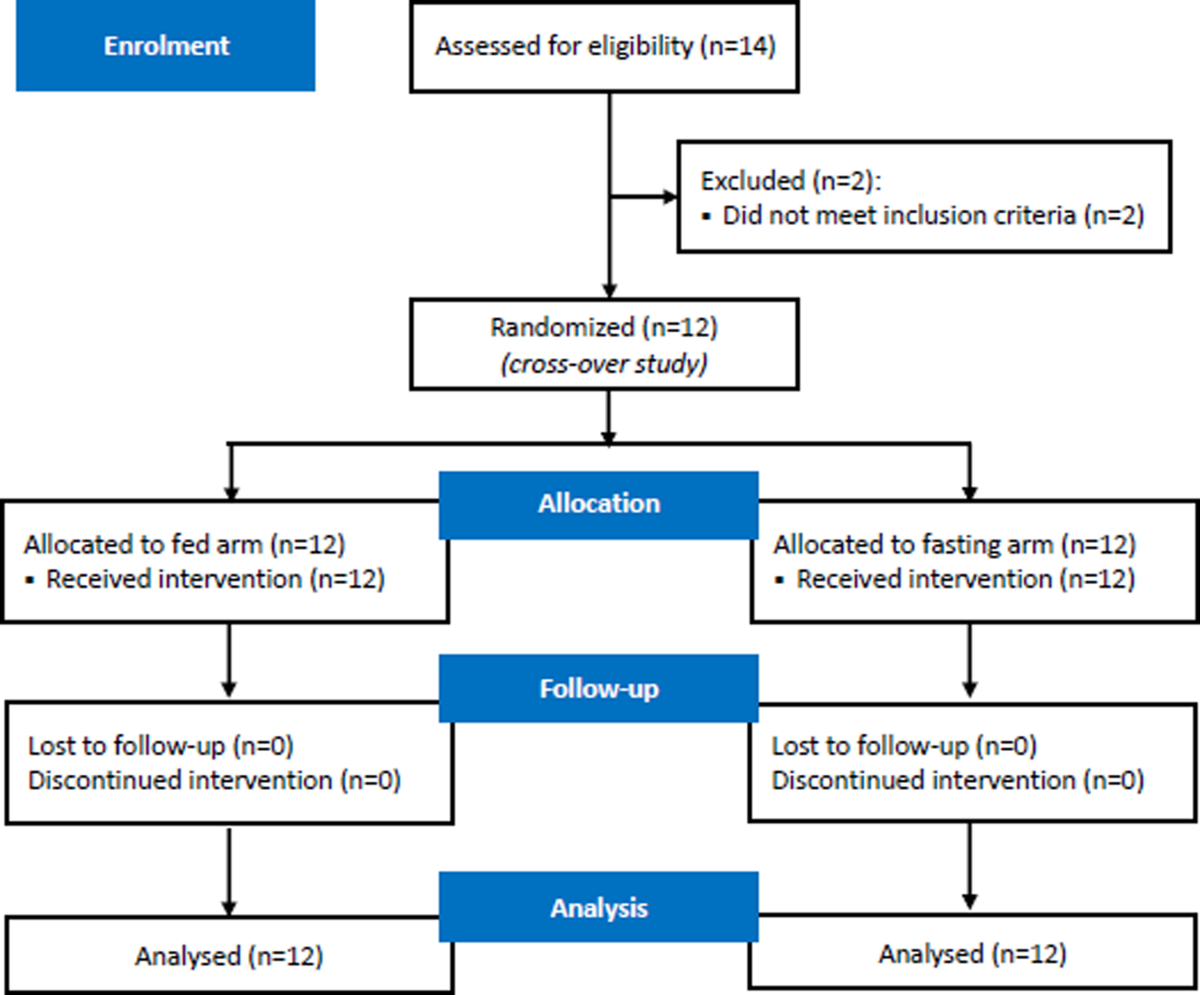
CONSORT flow diagram describing volunteer enrolment, allocation to intervention, follow-up and analysis.

The 12 participants (5 female, 7 male) had a mean (range) age of 26 (21–38) years, weight of 74 (54–96) kg and height of 176 (155–193) cm. All eligible volunteers randomized to intervention completed both study days and were included in analysis (Fig 1).

The full concentration-time data for plasma and urine are available in S1 Dataset.

### Non-compartmental pharmacokinetic analysis

**Fig 2:**
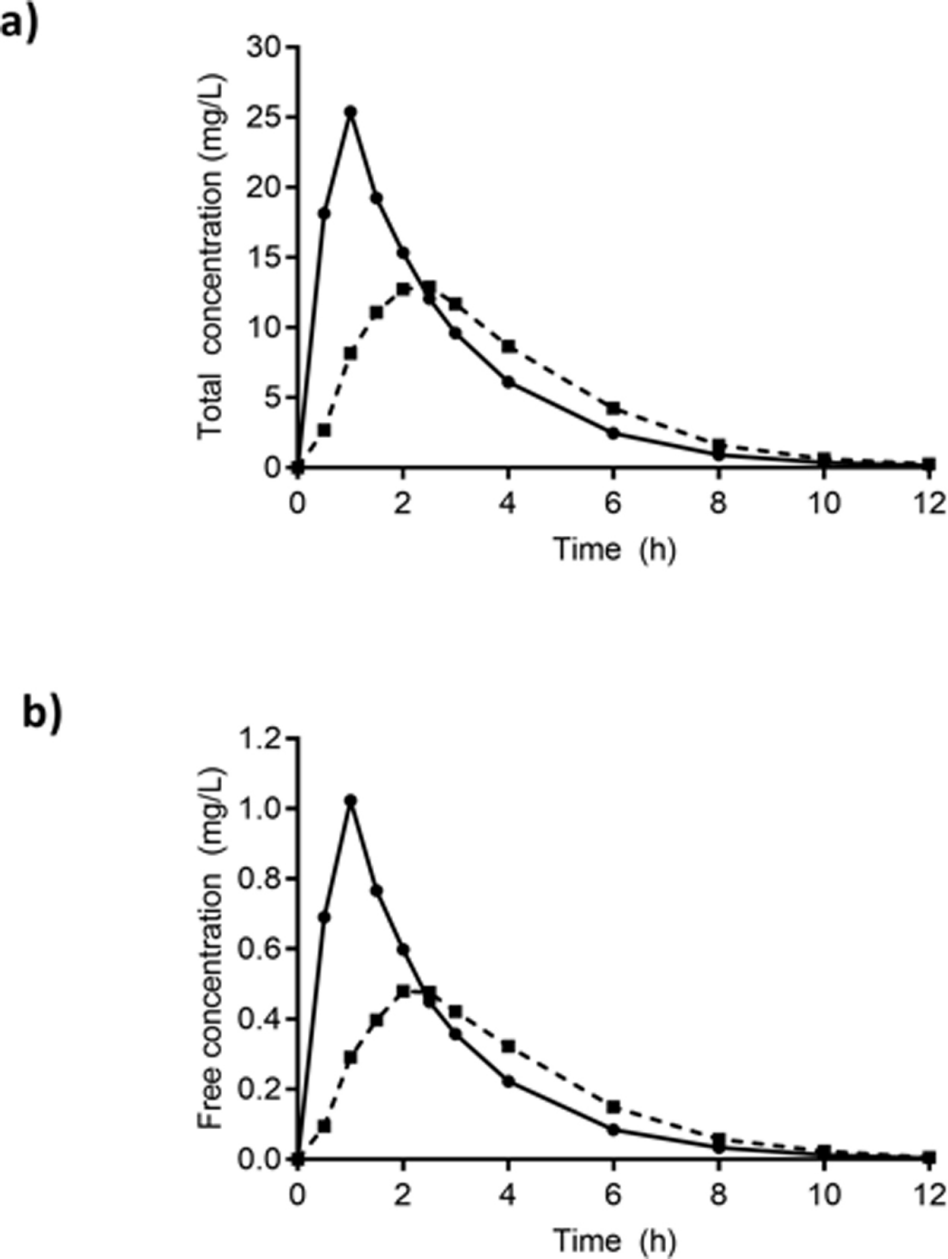
Curves of geometric mean concentrations for the whole group. (a) Total flucloxacillin concentrations, and (b) free flucloxacillin concentrations in the fed (■) and fasting state (●). Error bars are not shown since statistics were performed on paired differences.

Plasma concentration-time curves for geometric mean total and free flucloxacillin for the fed and fasting states are plotted in Fig 2. Values for pharmacokinetic parameters for total and free flucloxacillin concentrations for fed and fasting states are shown in Table 1, along with the within-subject changes with and without food ie. paired t-test of the ratio of the fed to fasting states.

**Table 1:** Mean (95% CI) values for the pharmacokinetic parameters for total and free plasma flucloxacillin concentrations in the fed and fasting states, and ratios^a^ of fed/fasting (n = 12) based on non-compartmental analysis.

	AUC_0-∞_	*C*_max_	*T*_max_	CL/F	t½
	(mg/L.h)	(mg/L)	(h)	(L/h)	(h)
**Total concentrations**					
Fed	59.8	14.8	2.23	16.7	1.54
	(53.1–67.5)	(12.6–17.4)	(1.84–2.62)	(14.8–18.9)	(1.37–1.71)
Fasting	71.6	27.4	0.97	14.0	1.58
	(55.8–90.8)	(20.4–36.8)	(0.8–1.14)	(11.0–17.8)	(1.47–1.71)
Ratio fed/fasting	0.84*	0.46***	2.37***	1.25 NI	0.96 BE
	(0.71–0.98)	(0.37–0.57)	(2.01–2.63)	(1.02–1.4)	(0.86–1.08)
**Free concentrations**					
Fed	2.23	0.55	2.33	448	1.57
	(1.86–2.69)	(0.43–0.71)	(1.97–2.70)	(372–539)	(1.40–1.75)
Fasting	2.79	1.08	1.08	359	1.50
	(2.23–3.47)	(0.81–1.43)	(0.96–1.21)	(288–448)	(1.32–1.69)
Ratio fed/fasting	0.80**	0.51***	2.21***	1.25*	1.05 BE
	(0.70–0.92)	(0.42–0.62)	(1.87–2.55)	(1.08–1.44)	(0.92–1.19)

^a^Ratios represent within-subject comparisons (means of differences compared by paired t-test, with 90% CI as is standard for bioequivalence testing).

NI = non-inferior, BE = bioequivalent

* = p<0.05,

** = p<0.01,

*** = p<0.001

### Non-compartmental pharmacokinetic analysis

For total plasma concentrations of flucloxacillin, the mean fed/fasting AUC_0-∞_ ratio of 0.84 was consistent with the ratio of 0.81 for the amount excreted in urine for 12 hours post-dose in fed versus fasting states (mean of 387 mg with food versus 476 mg in the fasting state). The different shapes of the concentration-time curves of fed vs fasting was explained by the reduced *C*_max_ and longer *T*_max_ in the fed state.

The shapes of the curves for free plasma concentrations of flucloxacillin were very similar to those of total flucloxacillin, consistent with the mean value of protein binding for the fed and fasting states combined of 0.963 (95% CI 0.955–0.970). The protein binding was not different between treatment regimens, with a mean fed/fasting ratio of 1.01 (90% CI 0.98–1.02), The values of fed/fasting *C*_max_ and *T*_max_ ratios for free concentrations were very similar to those of total concentrations. There was no difference in free *t*_1/2_ between fed and fasting states, and this was bioequivalent for both total and free concentrations.

### Pharmacokinetic and pharmacodynamic modelling

A two-compartment model was excluded because of no suggestive visual evidence in the individual concentration-time curves, and by higher values for the Akaike information criteria (AIC) compared with a one-compartment model using PKSolver. There was clear evidence of a *T*_lag_, both in the appearance of the concentration-time curves and by lower AIC values (S2 Dataset). There was little difference between first-order and zero-order absorption (S3 Dataset and S1 Fig), with a tendency for first-order to fit better for the fasting data, and zero-order for the fed data. For simultaneous modelling of the fed and fasting data, we chose first-order absorption because this was more likely *a priori*. The PTA curves based on first-order absorption were almost superimposable with those based on zero-order absorption. The mean values (and 95% CI) for the parameters of the chosen model in the fed and fasting states are shown in Table 2. The 95% CIs include both inter-individual and inter-occasion variability. The values for free volume of distribution (V/F) and for free clearance (derived from CL/F = V/F x *k*_el_ in Table 2) in the fasting and fed states were similar to the respective values from our non-compartmental analysis (Table 1), and to published values in healthy patients, when corrected for oral availability (0.5) and protein binding (0.95) [11]. These results support the validity of the model and show that the major effects of food were an increase in the *T*_lag_ and a decrease in the rate of absortion.

**Table 2:** Population parameter values (95% CI) for free concentrations, modelled with Monolix using a one-compartment model with *T*_lag_.

	*T*_lag_ (h)	*k*_a_ (h^-1^)	V/F (L)	*k*_el_ (h^-1^)
Fed	0.35	0.74	822	0.55
	(0.15–0.82)	(0.15–3.8)	(418–1617)	(0.35–0.85)
Fasting	0.19	3.6	670	0.53
	(0.08–0.45)	(0.71–18.2)	(341–1317)	(0.34–0.83)

*k*_a_, absorption rate constant; *k*_el_, elimination rate constant; V/F, apparent oral volume of distribution where F is oral availability.

**Fig 3:**
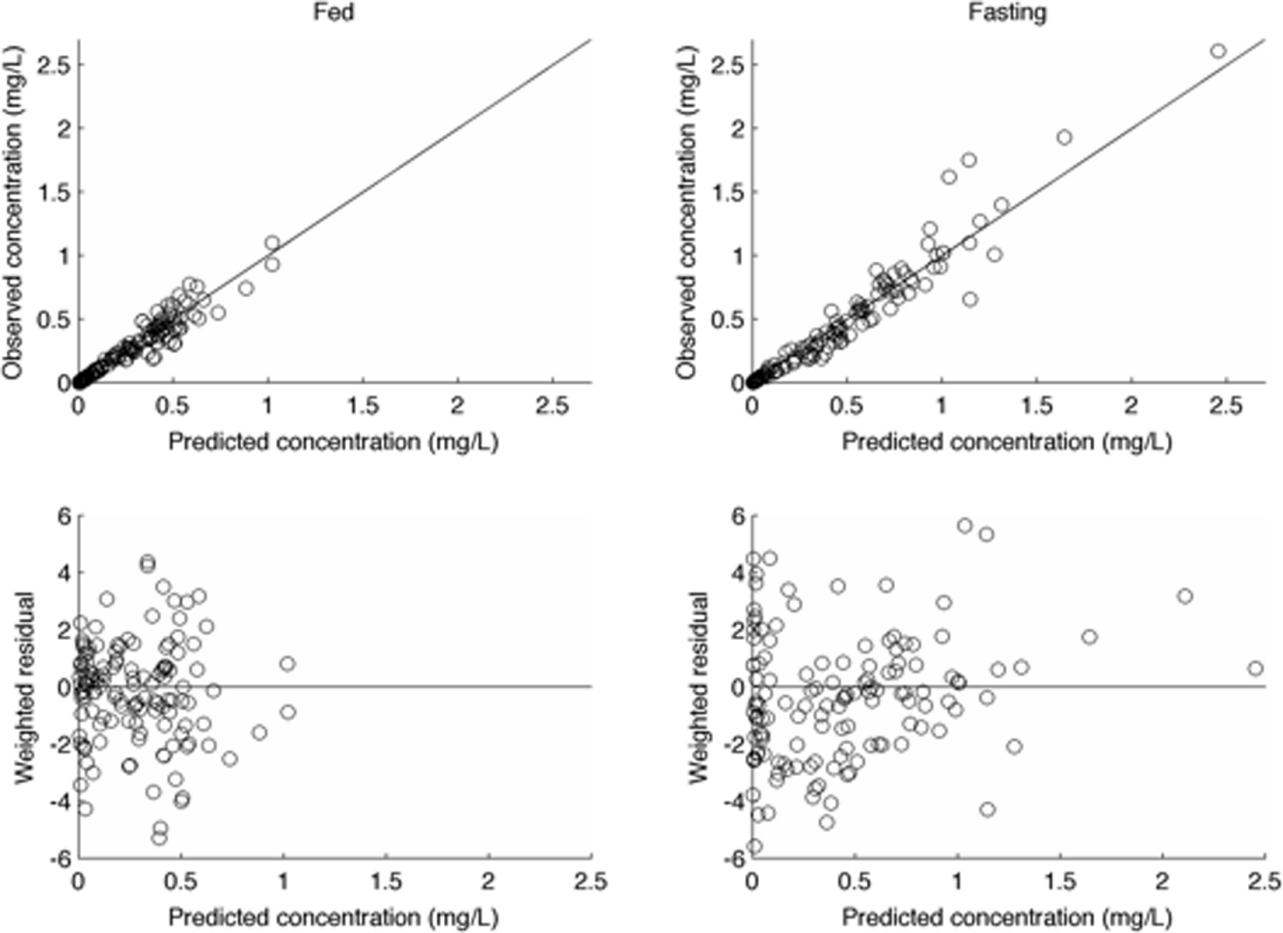
Regression and residual plots of observed versus predicted concentrations in the fed (r = 0.96) and fasting (r = 0.97) states. Weighted residuals represent the number of standard deviations by which each observation differs from the predicted value according to the normally distributed error model used.

Free plasma concentrations of flucloxacillin exceeded for 30%, 50% and 70% of the first 6 and 8 hours after dosing are shown in Table 3 for the raw data and for 6- and 8-hour dose intervals at steady-state for the modelled data. The observed versus model-predicted concentration data are shown in Fig 3. These show a Pearson’s correlation coefficient (r) of 0.96 for the fed state and 0.97 for the fasting state, with an even distribution of weighted residuals confirming accuracy and precision of the model.

**Table 3:** Free flucloxacillin concentrations (geometric means) exceeded for 30%, 50% and 70% of 6- and 8-hour dose intervals in the fed and fasting states. The ratio fed/fasting (geometric mean ± 90% CI) following 1000 mg oral flucloxacillin is also provided based on raw data, and modelled to steady-state.

Dose interval	Fed	Fasting	Fed/Fasting^a^
	Mean concentration (mg/L)	Mean concentration (mg/L)	Mean
	(95% CI)	(95% CI)	(90% CI)
a) Initial dose, 30% of			
6 hour	0.39	0.53	0.73 **
	(0.33–0.46)	(0.42–0.67)	(0.62–0.86)
8 hour	0.34	0.40	0.83
	(0.28–0.44)	(0.32–0.52)	(0.73–0.96)
b) Initial dose, 50% of			
6 hour	0.29	0.31	0.95 BE
	(0.24–0.36)	(0.24–0.40)	(0.82–1.10)
8 hour	0.23	0.20	1.11 NI
	(0.18–0.28)	(0.16–0.26)	(0.94–1.31)
c) Initial dose, 70% of			
6 hour	0.21	0.19	1.14 NI
	(0.17–0.26)	(0.15–0.23)	(0.96–1.35)
8 hour	0.14	0.10	1.39 *
	(0.11–0.17)	(0.08–0.13)	(1.10–1.75)
d) Steady-state, 30% of			
6 hour	0.48	0.64	0.74 **
	(0.39–0.59)	(0.53–0.78)	(0.65–0.85)
8 hour	0.38	0.48	0.81 *
	(0.31–0.47)	(0.40–0.57)	(0.70–0.93)
e) Steady-state, 50% of			
6 hour	0.36	0.37	0.97 BE
	(0.30–0.44)	(0.31–0.45)	(0.85–1.12)
8 hour	0.25	0.22	1.13 NI
	(0.21–0.30)	(0.18–0.27)	(0.96–1.32)
f) Steady-state, 70% of			
6 hour	0.25	0.21	1.23 NI
	(0.21–0.31)	(0.17–0.26)	(1.02–1.48)
8 hour	0.14	0.10	1.48*
	(0.11–0.18)	(0.07–0.13)	(1.15–1.89)

^a^Ratios represent within-subject comparisons (means of differences compared by paired t-test, with 90% CI as is standard for bioequivalence testing).

BE = bioequivalent, NI = non-inferior,

* p<0.05,

** p<0.01

The fed/fasting ratios of free concentrations exceeding 30% of the first 6 and 8 hours after the dose, and predicted at steady state, indicated statistical inferiority of the fed state by mean values of 0.73- to 0.83-fold. For free concentrations exceeding 50% of these targets the fed state was at least bioequivalent to the fasting state. For free concentrations exceeding 70% of these targets, the fed state was non-inferior or superior by mean values of 1.14- to 1.48-fold (Note: non-inferiority indicates at least bioequivalence with a trend in favour of the fed state).

**Fig 4:**
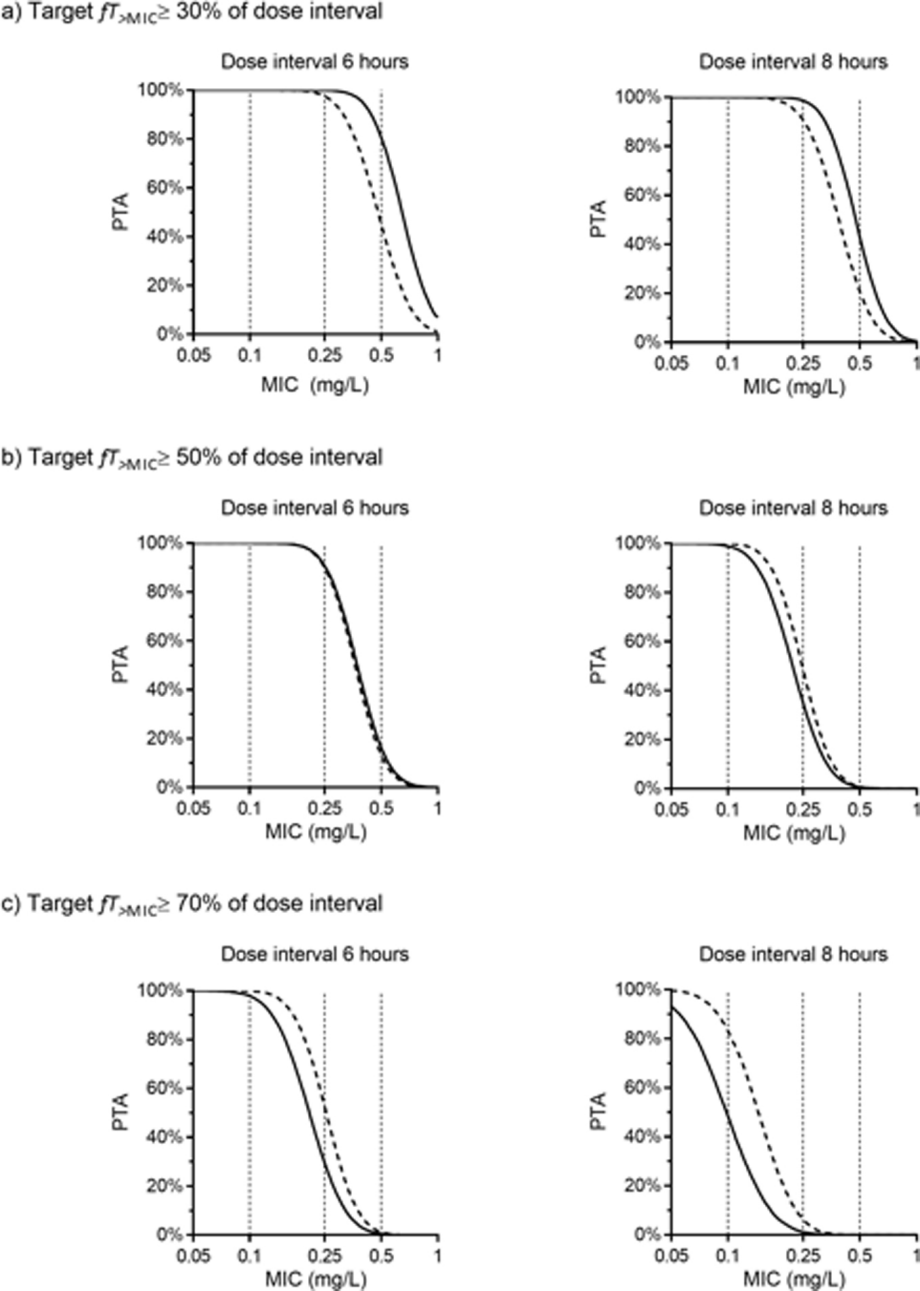
Probability of target attainment (PTA) for 30%, 50% and 70% of 6- and 8-hour dose intervals with flucloxacillin 1000 mg orally modelled to steady-state (one-compartment, first-order absorption with T_lag_) for a range of MICs. Serrated line: fed; continuous line: fasting. Relevant MIC_90_ of *S*. *pyogenes* (0.1 mg/L) and *S*. *aureus* (0.5 mg/L) are indicated by vertical serrated lines. Also shown is the MIC_50_ of *S*. *aureus* (0.25 mg/L).

The PTA simulations of free flucloxacillin concentrations exceeding 30%, 50% and 70% of a range of MIC values in the fed and fasting states, based on the 1000 mg dose modelled at steady-state, are illustrated in Fig 4. For a PTA for 30% of the dosing interval, food shifted the curve to the left for both 6- and 8-hour dosing. For a PTA for 50% of the dosing interval, food had no effect for 6-hour dosing and shifted the curve slightly to the right for 8-hour dosing. For a PTA for 70% of the dosing interval, food shifted the curve to the right for both 6- and 8-hour dosing. A shift to the left indicates superiority of the fasting state while a shift to the right indicates superiority of the fed state.

The PTAs for exceeding an MIC of 0.5 mg/L were inferior in the fed versus fasting state for the target of 30% of both 6 h and 8 h dose intervals, but almost identical for 50% and 70% targets. The PTAs for exceeding an MIC of 0.25 mg/L were very similar in the fed and fasting states for a target of 30% of both 6 h and 8 h dose intervals, and for a target of 50% of a 6 h dose interval. These PTAs were all greater than 90%. The PTAs for a target of 50% of an 8 h dose interval, and for 70% of both 6 h and 8 h dose intervals were superior in the fed state.

## Discussion

This study in volunteers investigated the impact of a standardized, high-fat, high-calorie meal on free flucloxacillin pharmacokinetics, using a sensitive assay, over the full concentration-time curve and with regard to contemporary pharmacodynamic targets. Flucloxacillin taken with food was associated with a significant reduction in free and total AUC and *C*_max_, and a prolonged *T*_max_, as shown with total flucloxacillin concentrations in previous studies [5,6]. These effects resulted from a decrease in oral availability, a longer *T*_lag_ and a lower *k*_a_. Differences in achievement of pharmacodynamic targets were minor and ranged from a small reduction in PTA for a free concentration target of greater than 0.5 mg/L for 30% of the dose interval, bioequivalence for a target of 50%, and non-inferiority for a target of 70%.

### Clinical implications

These results have important implications for clinical practice as they provide a scientific basis for reconsidering the recommendation that flucloxacillin should always be administered on an empty stomach. Our study shows that for mild to moderate skin and soft tissue infections caused by all β-haemolytic streptococci and most strains of *S*. *aureus*, food has no adverse effect on a pharmacodynamic target of bacteriostasis. For this patient group, bacteriostasis is probably all that is necessary. For the small subpopulation of patients infected with a strain of *S*. *aureus* with an MIC > 0.25 mg/L, the PTA simulations suggest a potential benefit of the fasting state, as there is a greater probability of achieving the target concentration for bacteriostasis (30% of the dose interval). It is often difficult to determine whether skin and soft tissue infections such as cellulitis are caused by *S*. *aureus* or β-haemolytic streptococci but other infections such as a boil or carbuncle are typically caused by *S*. *aureus* [20–22]. In either case the MIC of the pathogen is rarely known, so that the most certain approach in these conditions may be to recommend administration on an empty stomach. Nevertheless, this potential gain in a small proportion of cases must be balanced against the difficulty that many patients experience in remembering to take flucloxacillin an hour before meals and the likelihood of missed doses, which may have a much greater effect on the clinical efficacy. Additionally, if it was clinically important to reach a bactericidal target for all potentially infecting *S*. *aureus* strains, and for a larger percentage of the dose interval, as might be required in severe disease or in neutropenic sepsis, the fed state offers no disadvantage over the fasting state. In these situations, oral flucloxacillin in doses of 1000 mg 6-hourly, irrespective of whether it is taken with food or in the fasting state, may fail to achieve adequate therapeutic targets for many strains and an alternative treatment strategy should be considered. An increase in the oral flucloxacillin dose (beyond 1000 mg) or dosing frequency (e.g. 4 hourly) is usually impractical so the most likely consideration is parenteral flucloxacillin. Regardless of the effect of food, the results indicate that a dose of 1000 mg 6- or 8-hourly will achieve an adequate PTA for infections caused by almost all strains of β-haemolytic streptococci and *S*. *aureus*. This supports results from other volunteer studies [23].

The results were modelled for both 6- and 8-hourly dosing, as both regimens have been recommended, and because in practice it is almost impossible to dose precisely every 6 hours. At the least it is likely that there will be an 8-hour delay while the patient sleeps. The outcome was similar for these time intervals in that there was no difference in target attainment for an organism with an MIC of ≤0.25mg/L whether taken in the fed or fasting state. However, there was a lower probability of target attainment with 8-hourly dosing compared with 6-hourly dosing if the MIC was >0.25mg/L.

### Limitations

There are several limitations to this study. It was conducted with a fixed dose of 1000 mg of flucloxacillin in young, healthy volunteers with a relatively normal BMI and normal renal function. In practice, flucloxacillin is administered to patients with a wide range of age, BMI, and renal function [24–26]. Patients with impaired clearance of flucloxacillin will have an increase in the PTA, both with and without food, and this may increase the chance of therapeutic success. Additionally, the food given to the volunteers was a high-fat, high-calorie meal, and this may be larger than that usually consumed by patients. Any effect of administration with meals in clinical practice may well be less than demonstrated here with volunteers. We have not investigated the effect of food on doses of flucloxacillin other than 1000 mg, but it is likely that the effect would be similar. Finally, the steady-state data rests on the validity of the one compartment model with *T*_lag_ and first-order absorption. The use of this model was supported by the similarity of the free fed/fasting concentration ratios after the given dose and those at the predicted steady-state. This was expected since with a short *t*_½_ of flucloxacillin of around 1.5 h, steady state is closely approximated by the time a second dose is given at 6 or 8- h dose intervals. Because of the uncertainty of translating these findings into clinical practice it would be useful to study pharmacodynamic target attainment of flucloxacillin concentrations in the variety of clinical settings in which oral flucloxacillin is prescribed. These findings in adults may not translate directly into the paediatric population and this would be worthy of further study given that oral administration of flucloxacillin is particularly difficult in children [27]. Finally, it should not be assumed that the findings of this study extend to other isoxazolyl penicillins, but this too warrants further investigation.

## Conclusions

Contrary to what has been widely accepted since the 1970s, the results of this study indicate that taking flucloxacillin with food may not compromise effective flucloxacillin plasma concentrations in most circumstances. Other potential advantages of taking flucloxacillin with food include convenience, improved compliance and reduction in adverse effects such as nausea. Rather than focusing on administration on an empty stomach, it is more important that dosing is individualized according to known or suspected susceptibility to flucloxacillin, illness severity, and host characteristics. The findings of this study have significant implications for dosing of oral flucloxacillin worldwide.

## Supporting information

S1 Dataset Raw flucloxacillin urine concentration results fed versus fasting. (XLSX)

S2 Dataset Likelihood results for various combinations of *T*_lag_ versus no *T*_*l*ag_ and first- versus zero-order. (XLSX)

S3 Dataset Results for zero-order elimination. **For comparision with Fig 4 in paper.** Free flucloxacillin concentrations (geometric means) exceeded for 30%, 50% and 70% of 6- and 8-hour dose intervals in the fed and fasting state. The ratio fed/fasting (geometric mean ± 90% CI) following 1000 mg oral flucloxacillin is also provided based on raw data, and modelled to steady-state.
(XLSX)

S1 Fig For comparison with Fig 4 in the manuscript, which is also included below as a reference. Probability of target attainment (PTA) for 30%, 50% and 70% of 6- and 8-hour dose intervals with flucloxacillin 1000 mg orally modelled to steady-state using zero-order absorption for a range of MICs. Serrated line: fed; continuous line: fasting. Relevant MIC_90_ of *S*. *pyogenes* (0.1 mg/L) and *S*. *aureus* (0.5 mg/L) are indicated by vertical serrated lines. Also shown is the MIC_50_ of *S*. *aureus* (0.25 mg/L).
(DOCX)

S1 File Study protocol. (PDF)

S1 Table TREND checklist. (DOC)
