# Supplementary figures and images for: In healthy volunteers, taking flucloxacillin with food does not compromise effective plasma concentrations in most circumstances
Source: PLoS One. 2018 Jul 12;13(7):e0199370. doi: 10.1371/journal.pone.0199370 (PMC6042703; doi:10.1371/journal.pone.0199370)

**S1 Fig**


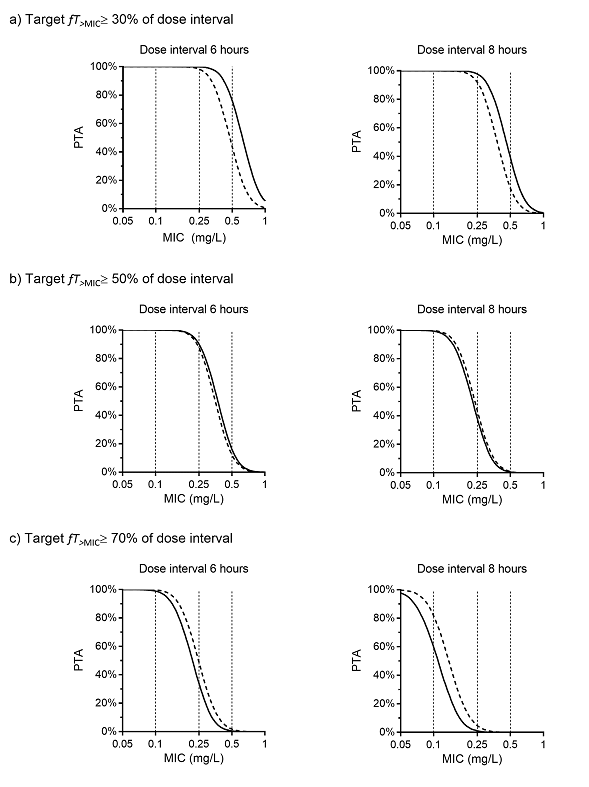


**Fig 3 (as in manuscript)**


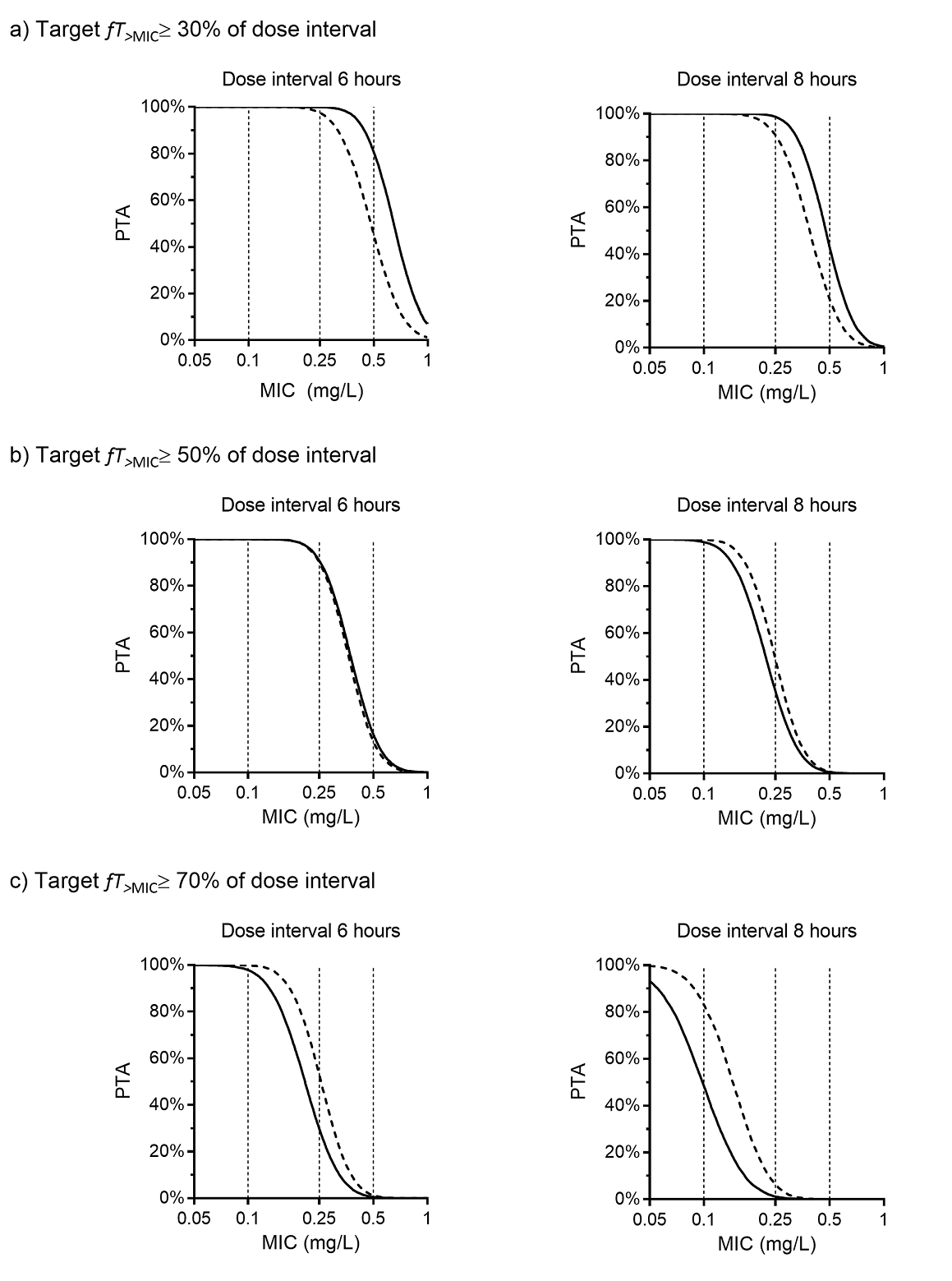

Supplement: S1 Fig — Probability of target attainment (PTA) for 30%, 50% and 70% of 6- and 8-hour dose intervals with flucloxacillin 1000 mg orally modelled to steady-state using zero-order absorption for a range of MICs. Serrated line: fed; continuous line: fasting. Relevant MIC90 of S. pyogenes (0.1 mg/L) and S. aureus (0.5 mg/L) are indicated by vertical serrated lines. Also shown is the MIC50 of S. aureus (0.25 mg/L). (DOCX) [file pone.0199370.s004.docx]
