# Supplementary material for: In healthy volunteers, taking flucloxacillin with food does not compromise effective plasma concentrations in most circumstances
Source: PLoS One. 2018 Jul 12;13(7):e0199370. doi: 10.1371/journal.pone.0199370 (PMC6042703; doi:10.1371/journal.pone.0199370)
Supplement: S1 File — (PDF) [file pone.0199370.s005.pdf]

Canterbury DHB
District Health Board
Te Poari Hauora ō Waitaha

UNIVERSITY of OTAGO
Te Whare Wānanga o Otāgo

Effect of food on flucloxacillin concentrations in volunteers

Protocol

Recruitment and consent

Recruit and consent 12 healthy volunteers aged 18 to 40 years who meet the following criteria:

- Taking no regular oral medications, except the oral contraceptive and asthma inhalers,
- eGFR > 80 mL/min,
- Have no health conditions (like Crohns or Coeliac disease) that might affect absorption of medication or cause intolerance of a standard high-fat meal (croissants, bacon and cheese) or that might make it difficult to go without food for up to 10 h (like type 1 diabetes),
- Have no contraindication to flucloxacillin, in particular:
    - no previous allergic reaction or other side effect to a penicillin- or cephalosporin-group antibiotic (including flucloxacillin-induced hepatitis),
    - not pregnant,
    - do not have severe liver disease.

Volunteers are sought through word of mouth and advertising within the Christchurch Hospital, especially the University of Otago - Christchurch. Volunteers are provided with information sheets beforehand on the trial and the medications involved. Each volunteer will sign a consent form for the study.

Each volunteer will fill out a questionnaire including information about health status and demographics. They will have measurements of weight and height and a physical examination including pulse and blood pressure. Blood tests for renal function, electrolytes and full blood count will be performed.

Plan for two study days

The 12 volunteers will each undertake the study on two days, separated by approximately one week. On these study days, the volunteers will take either:

1. Flucloxacillin 1000 mg orally on an empty stomach after an overnight fast, or
2. Flucloxacillin 1000 mg orally with a standardised breakfast after an overnight fast.

If there is a technical problem with study processes then one or two volunteers may need to repeat a study day. The order of study day fed versus fasting will be randomised (as if by the flip of a coin).

Preparation

Volunteers will attend the Nicholls Research Centre at Christchurch Hospital at 0730 h after an overnight fast (no food from 2200 h the night prior; water only allowed up until admission to the unit at 0730 h). Each volunteer will consent to take part and one of the study doctors will confirm that they are still fit to take part.

Meals

Fasting: Following an overnight fast of at least 10 h (from 2200 h the night prior), volunteers will ingest 2 x flucloxacillin 500mg capsules (1000 mg) with 240 mL of water at 0830 h. No food will be consumed for 4 h post dose. Water may be consumed from 1 h post dose. Caffeine may be consumed from 4 h post-dose. No alcohol is allowed during the study day.

Fed: Following an overnight fast of at least 10 h (from 2200 h the night prior), volunteers will commence eating a standardised meal (croissants, bacon and cheese) at 0800 h. The meal must be completed within

30 minutes. At 0830 h (30 minutes after the start of the meal), 2 x flucloxacillin 500 mg capsules (1000 mg) will be consumed with 240 mL of water. No further food will be allowed for at least 4 h post dose. Water may be consumed from 1 h post dose. Caffeine may be consumed from 4 h post-dose. No alcohol is allowed during the study day.

Blood tests

An intravenous (IV) catheter will be inserted and at ~0830 h (immediately prior to administration of 2 x flucloxacillin 500 mg capsules) a baseline blood test will be taken for flucloxacillin concentrations. The time of this blood test represents the start of the 12-h study period. Blood tests for flucloxacillin concentrations will then be done at 0.5, 1, 1.5, 2, 2.5, 3, 4, 6, 8, 10 and 12 h post-dose.

Each blood test will require withdrawal of ~5 mL of blood through the IV catheter (for discarding) then collection of a ~10 mL sample into purple-topped (2 x 4.5mL EDTA) tubes. The tubes will be centrifuged promptly and the plasma removed by pipette and stored -80°C until analysis in the Toxicology Department at Canterbury Health Laboratories. The IV catheter will be locked with normal saline between tests. The total volume of blood required from the volunteers will be ~180 mL on each study day.

Volunteers will document any side effects during the study day. The volunteers will be allowed to leave the Research Centre between tests in the afternoon.

Urine tests

Volunteers will be asked to void the bladder of urine at ~0830 h, just prior to flucloxacillin dose administration. All urine produced between the time of flucloxacillin administration and the final blood sample (~2030 h) will be collected so that the volume may be recorded and an aliquot taken for determination of flucloxacillin concentrations. This will help determine the completeness of flucloxacillin excretion from the body.

Concentrations and data analysis

The plasma samples will be analysed for total (bound + unbound) and free (unbound) flucloxacillin concentrations. Pharmacokinetic parameters including area under the concentration-time curve (AUC), time to peak concentration (Tmax), peak concentration (Cmax) and elimination half-life ($t_{1/2}$) will be determined for each subject on each study day.
